# The First Reported Case of Treating the Ultra-Central Thorax With Cone Beam Computed Tomography-Guided Stereotactic Adaptive Radiotherapy (CT-STAR)

**DOI:** 10.7759/cureus.62906

**Published:** 2024-06-22

**Authors:** Stephanie Zhao, Robbie Beckert, Xiaodong Zhao, Eric Laugeman, Clifford G Robinson, Gregory Vlacich, Pamela P Samson, Joshua P Schiff

**Affiliations:** 1 Department of Radiation Oncology, Washington University School of Medicine, St. Louis, USA

**Keywords:** image-guided radiotherapy, motion management, online adaptive radiotherapy, non-small cell lung cancer (nsclc), interfraction motion, sbrt (stereotactic body radiotherapy)

## Abstract

Stereotactic body radiotherapy (SBRT) to the central and ultra-central thorax is associated with infrequent but potentially serious adverse events. Adaptive SBRT, which provides more precise treatment planning and inter-fraction motion management, may allow the delivery of ablative doses to ultra-central tumors with effective local control and improved toxicity profiles. Herein, we describe the first reported case of cone beam computed tomography (CBCT)-guided stereotactic adaptive radiotherapy (CT-STAR) in the treatment of ultra-central non-small cell lung cancer (NSCLC) in a prospective clinical trial (NCT05785845). An 80-year-old man with radiographically diagnosed early-stage NSCLC presented for definitive management of an enlarging ultra-central lung nodule. He was prescribed 55 Gy in five fractions with CT-STAR. A simulation was performed using four-dimensional CT, and patients were planned for treatment at end-exhale breath-hold. Treatment plans were generated using a strict isotoxicity approach, which prioritized organ at risk (OAR) constraints over target coverage. During treatment, daily CBCTs were acquired and used to generate adapted contours and treatment plans based on the patient’s anatomy-of-the-day, all while the patient was on the treatment table. The initial and adapted plans were compared using dose-volume histograms, and the superior plan was selected for treatment. The adapted plan was deemed superior and used for treatment in three out of five fractions. The adapted plan provided improved target coverage in two fractions and resolved an OAR hard constraint violation in one fraction. We report the successful treatment of a patient with ultra-central NSCLC utilizing CT-STAR. This case report builds on previously published in silico data to support the viability and dosimetric advantages of CT-STAR in the ablative treatment of this challenging tumor location. Further data are needed to confirm the toxicity and efficacy of this technique.

## Introduction

Lung cancer represents the leading cause of cancer mortality in the US and worldwide [1]. For non-small cell lung cancer (NSCLC) patients diagnosed with localized, early-stage disease (stages I-IIA), surgical resection with mediastinal lymph node dissection remains the cornerstone of treatment. Stereotactic body radiotherapy (SBRT) is an effective treatment of early-stage NSCLC in inoperable or high-risk operable patients, with three-year local control rates between 85% and 96% [2-5]. Optimal local control is predicated on the delivery of a biologically effective dose (BED10; alpha/beta = 10) of at least 100 Gy [4].

While SBRT for early-stage NSCLC has widely been a successful endeavor, the delivery of SBRT with optimal BED10 for patients with central and ultra-central lung tumors has historically proven to be challenging. Multiple studies have demonstrated high rates of grade ≥ 3 toxicity when treating the ultra-central thorax, and few patients experienced treatment-related death, most commonly secondary to bronchopulmonary hemorrhage [6,7]. Thus, radiation oncologists often treat with hypofractionated regimens for these patients (typically 60 Gy in eight to 15 fractions) to mitigate this toxicity. However, this often comes at the expense of a BED10 less than 100 Gy and does not necessarily translate to reduced toxicity [6,8].

Adaptive radiotherapy has emerged as a promising avenue to minimize toxicity to organs at risk (OARs) while delivering ablative radiotherapy doses. In adaptive radiotherapy, treatment plans are adjusted on a per-fraction basis based on the patient’s anatomy-of-the-day, all while the patient is on the treatment table, thereby allowing for inter-fraction motion management. Several studies have demonstrated that stereotactic MRI-guided adaptive radiotherapy (SMART) can allow for the delivery of high BED10 SBRT treatments to the ultra-central thorax [9-11]. More recently, in silico testing on a ring gantry cone beam computed tomography (CBCT)-based radiotherapy unit capable of conducting adaptive radiotherapy suggests that CBCT-guided adaptive radiotherapy may be dosimetrically safe and feasible for patients with ultra-central thoracic disease [12]. Herein, we describe the first reported case of the use of CBCT-guided stereotactic adaptive radiotherapy (CT-STAR) in the treatment of a patient with a radiographically diagnosed, early-stage ultra-central NSCLC in a prospective clinical trial.

## Case presentation

Baseline patient information

**Figure 1:**
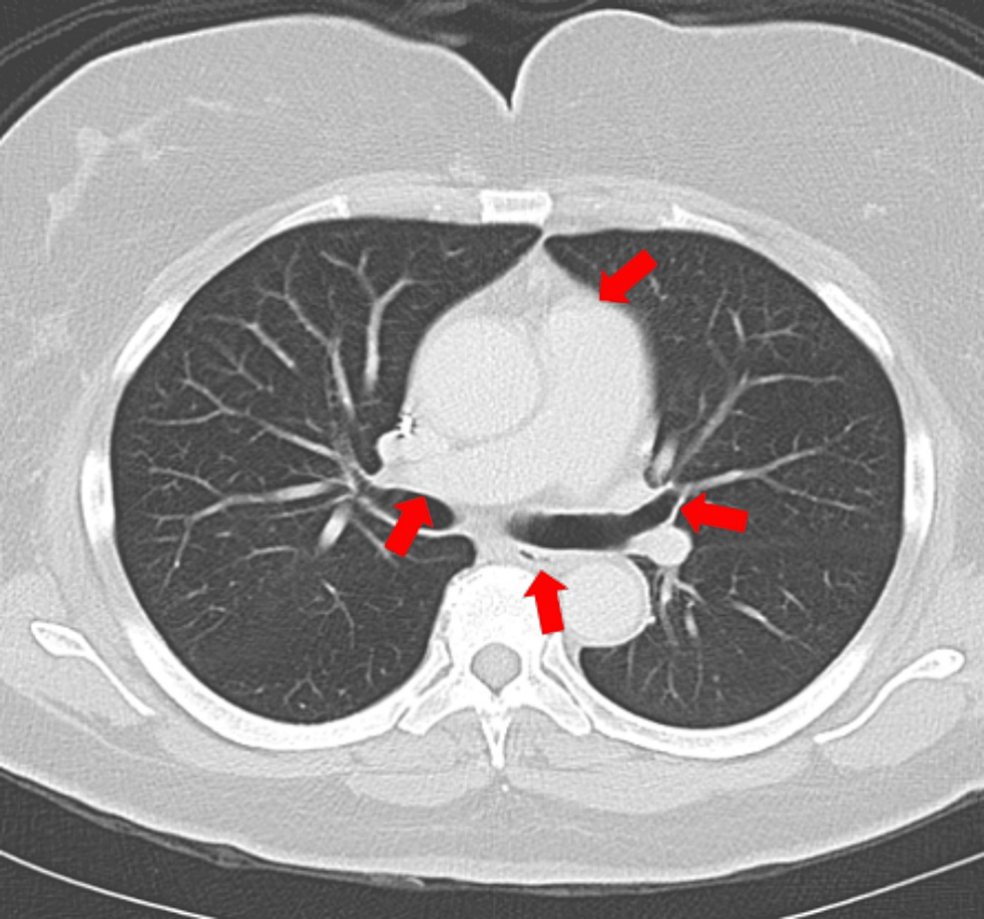
Ultra-central tumor locations Example locations of ultra-central thoracic tumors illustrated on a sample chest CT, with arrows pointing to the heart, right pulmonary artery, left proximal bronchial tree, and esophagus.

**Figure 2:**
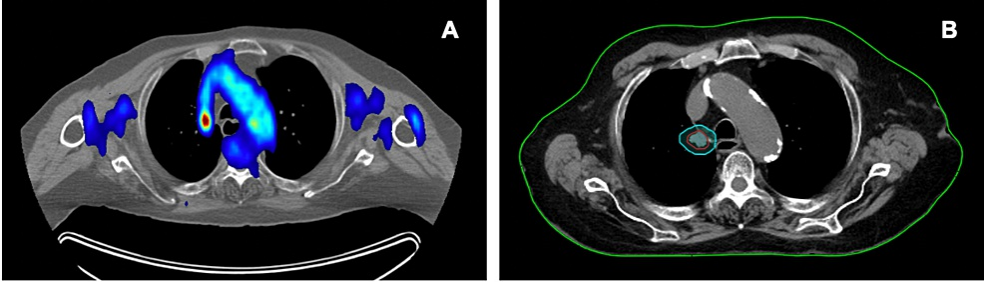
Tumor location and target contours Location of ultra-central lung nodule on PET/CT (A) at time of decision to undergo treatment. Target contours with gross tumor volume (GTV) in red and planning target volume (PTV) in cyan (B).

An 80-year-old man with a 40-pack-year smoking history first presented to radiation oncology for consultation of a slowly enlarging lung nodule incidentally discovered on chest CT and surveilled on imaging for two years due to patient preference. The nodule was located in the right upper lobe in close proximity to the trachea and esophagus and, therefore, classified as an ultra-central lesion (Figures 1-2). Given his age, multiple medical comorbidities, and ultra-central location of the tumor, the patient declined upfront SBRT and opted for continued short-term imaging follow-up of the nodule. An attempt for biopsy via endoscopic ultrasound was unsuccessful. Two years later, the enlarging nodule measured 2.0 × 1.3 cm with an interval increase in 18-fluoro-deoxyglucose positron emission tomography (FDG-PET) avidity. After discussion with the multidisciplinary team, the patient was recommended empiric treatment of his radiographically diagnosed, early-stage ultra-central NSCLC with 55 Gy in five fractions with CT-STAR on a phase 1 clinical trial (NCT05785845).

Clinical trial details

NCT05785845 is a prospective single-institution phase 1 clinical trial evaluating the toxicity of 55 Gy in five fractions CT-STAR for patients with biopsy-proven or radiographically diagnosed high-risk central (within 1 cm of the proximal bronchial tree) and ultra-central early-stage NSCLC. The early-stage disease includes patients with stages I-IIA disease, excluding tumors invading through the bronchial tree or great vessels, and patients who could not have prior radiation in the projected treatment field. All patients will be treated on the Ethos system (Varian Medical Systems, Palo Alto, CA), which is our institution’s CBCT-guided adaptive radiotherapy unit. The primary outcome of this study is acute grade ≥ 3 toxicity at one year, which is being compared to a historical control of 20%. Exploratory outcomes include toxicity at the two- and three-year time points as well as local control, progression-free survival, and overall survival. The patient described in this case report was the first patient treated in this study.

Treatment planning and delivery

**Figure 3:**
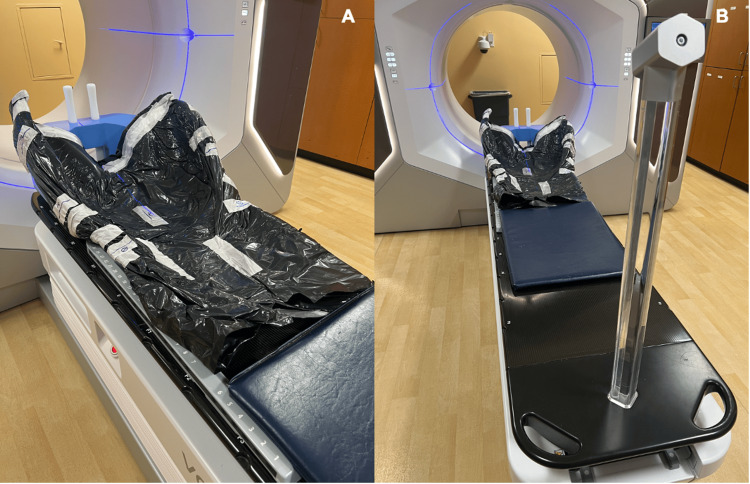
Custom immobilization device Two different images (A, B) of a custom immobilization device used for treatment on this study. Note the device allows for the patients arms to be placed overhead while immobilizing the patient.

The patient was simulated with end-exhale breath-hold CT and four-dimensional CT. A custom immobilization device (Figure 3) was used to position the patient supine with both arms overhead. At the time of simulation, a CBCT scan was obtained on the Ethos system to assess the patient’s breath hold capability and image quality of the CBCT. Gross tumor volume (GTV) was identified using simulation and diagnostic imaging. No clinical target volume was used to keep with standard institutional SBRT policy. A 5-mm expansion from the GTV was used to delineate the planning target volume (PTV). Intra-fraction motion management was conducted via end-exhale breath-hold using a surface-guided monitoring system, which has previously been described [13]; therefore, an internal target volume was not used.

Dose constraints for critical thoracic OARs, including the trachea, proximal bronchial tree, great vessels, heart, esophagus, brachial plexus, and spinal cord, are listed in Table 1. Both initial and adapted plans were generated under a strict isotoxicity approach, as previously described [14], which prioritized OAR constraints over target coverage. Per trial protocol, a D98% of greater than 45 Gy must be achieved for the GTV in order to maintain minimum appropriate coverage of the target, and if that metric is not met, then the patient will be screen-failed and treated off-trial. This metric was selected after evaluation of our in silico data in which minimum coverage was inadequate with CT-STAR in certain patients, and 45 Gy in 5 fractions is approximately equivalent to the BED10 of the 10-12 fraction regimen a patient would receive off trial at our institution. A planning optimization structure (PTV_Opt) was generated by subtracting OARs plus gradient margins from the PTV, which was used to deprioritize target coverage in areas of PTV and OAR overlap. A gradient margin of 3 mm was used for the trachea, bronchial tree, and great vessels, while a 5 mm gradient margin was used for the esophagus, heart, and brachial plexus.

**Table 1:** Target and OAR metrics Target volume metrics and organ at risk (OAR) constraints applied to both initial and adapted plans.

Target volume	Metrics required to be met during adaptation
PTV_5500	The maximum dose will be located within the target and outside the areas of OAR gradient margins used for planning.
GTV_5500	D 98% > 45 Gy
Target volume	Metrics not required to be met during adaptation
PTV_5500	V 95% Rx > 95% D 0.03 cc < 130%
PTV_Opt	V 55 Gy > 100%
Organ at risk	Metrics required to be met during adaptation
Trachea	V 50 Gy < 0.2 cc
Proximal bronchial tree	V 50 Gy < 0.2 cc
Esophagus	V 32 Gy < 0.5 cc
Stomach	V 33 Gy < 0.5 cc
Heart	V 32 Gy < 15 cc
Great vessels	V 47 Gy < 10 cc
Brachial plexus	V 27 Gy < 3 cc
Spinal cord	V 25 Gy < 1 cc
Organ at risk	Metrics not required to be met during adaptation
Chest wall	D max < 110% Rx
Uninvolved lung	Critical volume (CV) 13.5 Gy > 1000 cc CV 12.5 Gy > 1500 cc
Heart	D 0.03 cc < 105%
Great vessels	D 0.03 cc < 105%

Adapted plans were created based on the patient’s anatomy-of-the-day. Using a vendor-supplied artificial intelligence algorithm, the TPS automatically deformed and adjusted OAR contours onto the daily CBCT. The GTV generated during the planning CT was then rigidly copied onto the anatomy-of-the-day and edited at the discretion of the radiation oncologist and medical physicist, depending on changes in tumor and patient alignment. Then, the GTV and OARs within a contour ring were manually edited in real time to generate an adapted plan. All OARs within the contour ring that are deformed by the TPS are reviewed and edited as needed by the treating radiation oncologist during the online adaptive workflow prior to treatment delivery on every day of treatment. The initial (PI) and adapted (PA) plans were both evaluated on the patient’s anatomy-of-the-day and compared using dose-volume histograms. An adapted fraction was deemed superior and used for treatment if at least one OAR hard constraint was resolved when compared to the PI or if target coverage improved by 5% or greater when compared to the PI.

Dosimetric and treatment data

The patient underwent five fractions of CT-STAR without issue or evidence of acute toxicity. The median (range) PTV V55Gy across all five fractions was 85.9% (79.5-93.1) and 89.0% (83.1-91) in the PI and PA, respectively. For GTV V55Gy, the median (range) was 99.5% (97.0-99.9) for the PI and 99.7% (96.2-100.0) for the PA. Minimum coverage to 98% of the GTV was also evaluated, yielding median values of 11.87 Gy (10.6-12.3) in the PI and 11.67 Gy (10.0-12.3) in the PA.

**Figure 4:**
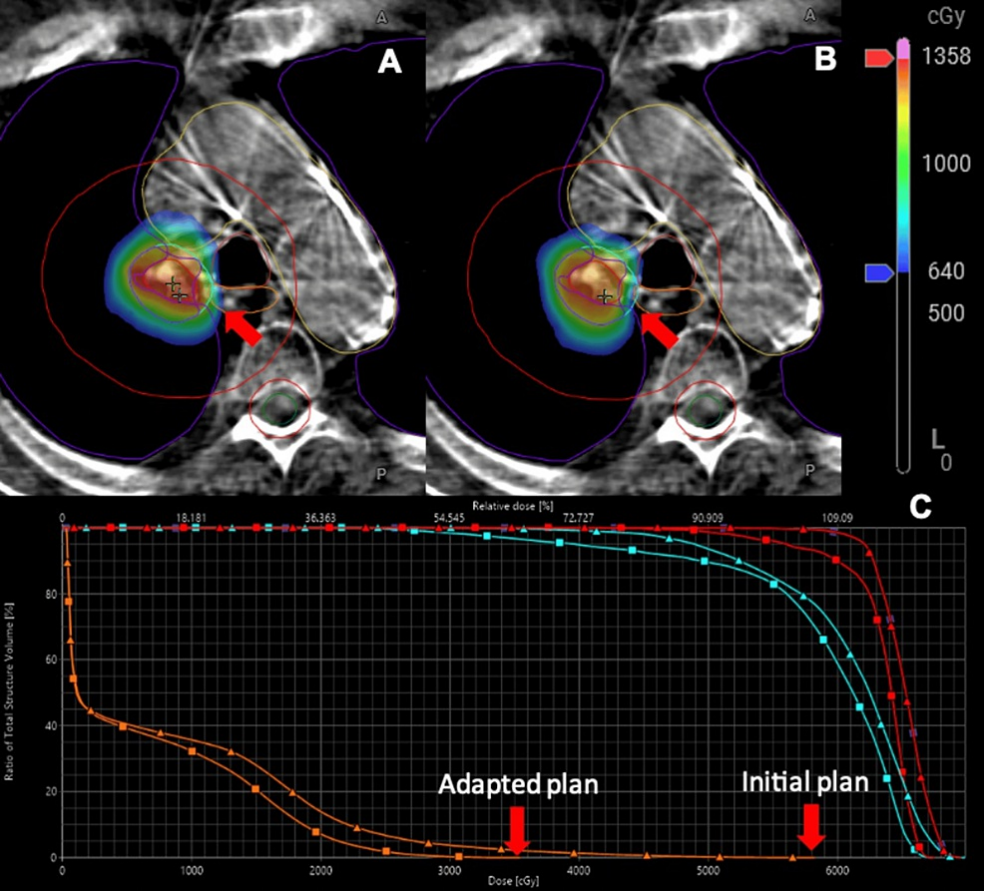
Initial and adapted plans Comparison of PI and PA using cone beam computed tomography (CBCT)-guided stereotactic adaptive radiotherapy (CT-STAR) for this patient with early-stage ultra-central non-small cell lung cancer (NSCLC). PI (A) and PA (B) for fraction three of treatment. In the PI, the esophageal constraint (V32Gy < 0.5cc) would have been violated, which was resolved using the PA. The dose to the esophagus, gross tumor volume (GTV) (red), and planning target volume (PTV) (cyan) are represented in the dose-volume histogram (DVH) (C) for both the PI (triangles) and PA (squares). Note that the dose on the CBCT images reflects the per-fraction dose, and the dose on the DVH represents the projected dose across all five fractions.

Out of five total fractions, the PA was selected over the PI in three fractions. In two of those fractions, the PA yielded a target coverage metric improvement of approximately 5%. In fraction two, the PTV V55Gy was improved from 83.00% to 89.10%, and in fraction four, the PTV V55Gy was improved from 79.50% to 84.30%. In fraction three, the PA was selected over the PI due to an esophagus constraint violation in the PI (Figure 4). In this fraction, the PA numerically reduced the esophagus V32Gy from 0.95 cc to 0.06 cc, meeting the constraint.

Timing data for each component of the adaptive planning and delivery process are displayed in Table 2. The mean total treatment time measured from the patient's entrance into the room was 67 minutes. The patient completed all treatments and is currently alive without evidence of high-grade toxicity or local recurrence at approximately one year post-treatment.

**Table 2:** Treatment time Treatment component times are listed in the table in minutes (min).

Fraction #	Patient enters room to initial CBCT acquisition (min)	Contouring (min)	Plan calculation (min)	Plan review (min)	Verification CBCT acquisition and review (min)	Delivery time (min)	Post-treatment scan acquisition and patient exit (min)	Total time (min)
1	46	11	7	6	8	11	4	93
2	11	15	6	4	13	8	7	64
3	9	16	5	2	12	9	5	58
4	8	18	5	3	14	10	4	62
5	13	11	6	1	7	14	6	58
Average	17	14	5	3	10	10	5	67

## Discussion

In this case report, we described the first reported use of CT-STAR for the delivery of ablative treatment to a patient with a radiographically diagnosed early-stage ultra-central NSCLC. Our results demonstrate improvements in target coverage greater than 5% for two fractions and resolution of an esophageal constraint violation in one fraction. The median percent volume of the PTV and GTV receiving this ablative dose (BED10 = 115.5 Gy) in both the PI and PA were greater than 85% and 99%, respectively. This case report provides further evidence supporting our prior in silico work on the feasibility and dosimetric advantages of CT-STAR for ultra-central thoracic disease [12].

The treatment of central and ultra-central lung tumors has often been associated with an increased risk of high-grade toxicity. When treated with 60-66 Gy in three fractions in the seminal report by Timmerman et al., patients with central tumors had an 11-fold higher risk of experiencing severe toxicity compared to patients with peripheral tumors [15]. While this risk has been moderated in subsequent studies using modified fractionation schedules [7], an ultra-central tumor location has remained a risk factor for significant toxicity, with rates of treatment-related death ranging from 0% to 22% [8,16]. A non-exhaustive compilation of ultra-central studies is displayed in Table 3. Recently, the phase II HILUS trial reported high rates of grade ≥ 3 toxicity (34%) and grade 5 toxicity (15%) following treatment of ultra-central tumors to 56 Gy in eight fractions [6]. The more recent phase I SUNSET study treated patients to 60 Gy in eight fractions (BED10 = 105.0Gy) and showed lower rates of grade ≥ 3 toxicity (6.7%) and grade 5 toxicity (3.3%). Compared to the HILUS trial, the SUNSET study excluded patients with tumors with endobronchial invasion, associated with a greater risk of hemorrhage, utilized a smaller PTV margin (5mm), and limited the radiation hotspot to 120% (compared to 150% in HILUS) of the prescribed dose [17]. Similarly, a meta-analysis highlighted continued variability in the planning of SBRT treatment for ultra-central tumors while balancing toxicity and effective local control [18]. Therefore, it remains challenging to deliver high-dose ablative SBRT to the ultra-central thorax with conventional treatment planning methods.

**Table 3:** SBRT for ultra-central disease in the literature Summary of studies published on treatment of ultra-central thoracic disease [6, 8, 11, 16-17, 19-23]. BED = biologically effective dose (alpha/beta=10); GTV = gross tumor volume; IMRT = intensity-modulated radiotherapy; N/R = not reported; PTV = planning target volume; SBRT = stereotactic body radiation therapy; SMART = stereotactic magnetic resonance-guided adaptive radiation therapy; VMAT = volumetric modulated arc therapy.

Author (year)	Definition of ultra-central	Dose/fraction	BED	Technique	Grade ≥ 3 toxicity at one year	Late Toxicity (grade ≥ 3)	Treatment related-death
Unger (2010) [19]	GTV -> trachea/proximal bronchial tree	30-40 Gy/5 fx	48-72 Gy	Cyberknife	N/R	15%	5%
Chaudhuri (2015) [20]	GTV -> trachea/proximal bronchial tree	50 Gy/5 fx	100 Gy	IMRT	0%	0%	0%
Tekatli (2016) [8]	PTV -> trachea or mainstem bronchus	60 Gy/12 fx	90 Gy	VMAT	20%	38%	21%
Haseltine (2016) [16]	GTV -> trachea/mainstem bronchus/lobar bronchus	45-50 Gy/5 fx	86-100 Gy	IMRT	20%	38%	22%
Lischalk (2016) [21]	GTV -> mainstem bronchus	35-40 Gy/5 fx	60-72 Gy	Cyberknife	N/R	10%	0%
Finazzi (2020) [22]	PTV -> trachea/proximal bronchial tree/pericardial pleura/mediastinal pleura	55 Gy/5 fx and 60 Gy/8 fx	115.5 Gy and 105 Gy	SMART	N/R	8%	0%
Lindberg (2021) [6]	GTV -> main bronchi/trachea	56 Gy/8 fx	95.2 Gy	SBRT	Grade 5 5%	34%	15%
Sandoval (2023) [11]	GTV -> main bronchi/trachea	54 Gy/3 fx, 50 Gy/5 fx, 60 Gy/5 fx, 60 Gy/8 fx, 50 Gy/10 fx, 60 Gy/15 fx	84-151.2 Gy	SMART	0%	4.3%	0%
Giuliani (2024) [17]	PTV -> bronchial tree/esophagus/pulmonary vessels	60 Gy/8 fx	105 Gy	SBRT	6.7%	N/R	3.3%
Levy (2024) [23]	PTV -> proximal bronchial tree/mediastinal or pericardial pleura	60 Gy/8 fx	105 Gy	SBRT	N/R	19.4%	6.4%

Notably, several studies have analyzed predictors of treatment-related death. In particular, the HILUS trial noted that a maximum dose of 0.2 cc of the main bronchi and trachea served as the strongest predictor of lethal bronchopulmonary hemorrhage [6]. This dependence of toxicity on critical thoracic OARs underscores the need for advanced treatment planning and motion management strategies, such as adaptive radiotherapy, to quantify and reduce the dose of OARs depending on the patient’s anatomy-of-the-day. Finazzi et al. illustrated the utility of SMART in the treatment of high-risk lung tumors; 50 lung tumors, including 30 tumors in a central location, were treated with a 95.6% rate of local control at 12 months, minimal grade 3 toxicity, and no grade 4 or 5 toxicities [19]. Furthermore, Sandoval et al. employed SMART to treat central and ultra-central thoracic lesions to ablative doses (median BED10 = 105Gy) with a similarly low toxicity profile [11]. Adaptive radiotherapy has, therefore, proven beneficial in treating ultra-central thoracic lesions safely and effectively and expanding the therapeutic index for SBRT in this tumor location. Unfortunately, SMART is not widely available; therefore, demonstrating the feasibility of adaptive radiotherapy to the ultra-central thorax using CBCT guidance is critical to increasing the accessibility of this technique to radiation oncology patients.

## Conclusions

Herein, we have described the first reported case of a patient with a radiographically diagnosed, early-stage ultra-central NSCLC treated with 55 Gy in five fractions using CT-STAR. This case report enhances our previously reported in silico data highlighting the dosimetric benefits of CT-STAR for this patient population. Further long-term follow-up of this patient and others treated on phase 1 clinical trial (NCT05785845) is needed to confirm whether these dosimetric benefits will confer clinical benefits such as reduced high-grade toxicity and high local control.
